# Increased Binding of Specificity Protein 1 to the *IL21R* Promoter in B Cells Results in Enhanced B Cell Responses in Rheumatoid Arthritis

**DOI:** 10.3389/fimmu.2018.01978

**Published:** 2018-09-05

**Authors:** Elizabeth M. Dam, Alison C. Maier, Anne M. Hocking, Jeffrey Carlin, Bernard Ng, Jane H. Buckner

**Affiliations:** ^1^Translational Research Program, Benaroya Research Institute, Seattle, WA, United States; ^2^Division of Rheumatology, Virginia Mason Medical Center, Seattle, WA, United States; ^3^Rheumatology Section, VA Puget Sound Health Care System, Seattle, WA, United States; ^4^Division of Rheumatology, Department of Medicine, University of Washington, Seattle, WA, United States

**Keywords:** rheumatoid arthritis, B cells, IL-21R, specificity protein 1, IL-6

## Abstract

B cells are implicated in rheumatoid arthritis (RA) based on the presence of autoantibodies and the therapeutic response to B cell depletion. IL-21 has a significant role in B cell development and function. Here we assess B cell responses to IL-21 and the mechanisms responsible for altered IL-21R expression in RA. Flow cytometry of PBMC and cultured B cells was used to quantify protein and mRNA levels of IL-21R, IL-21 signaling through pSTAT3, specificity protein 1 (SP1) and to determine cytokine production (IL-6) and maturation status of B cells in RA and healthy control subjects. SP1 binding to the *IL21R* promoter region in B cells was assessed with ChIP-qPCR. We demonstrate an increase in IL-21R expression in total and memory B cells from RA subjects, which correlated with responsiveness to IL-21 stimulation. Stimulation of naïve RA B cells with IL-21 and CD40L resulted in an increase in differentiation into plasmablasts and an increase in IL-6 production in comparison to healthy controls, which was dose dependent on IL-21 stimulation. IL-21R expression on memory B cells in RA synovial fluid was comparable to peripheral blood making our study pertinent to understanding B cell responses in the joint and site of inflammation. We identified an increase in SP1 protein and mRNA in RA B cells and demonstrate an increase in binding of SP1 to the *IL21R* promoter region, which suggests a mechanism by which IL-21R expression is enhanced on B cells in RA. Taken together, our results indicate a mechanism by which IL-21 enhances B cell development and function in RA through an SP1 mediated increase in IL-21R expression on B cells.

## Introduction

Rheumatoid arthritis (RA) is a chronic inflammatory and destructive joint disease characterized by the presence of rheumatoid factor and anti-cyclic citrullinated peptide autoantibodies (ACPA) (1, 2). Although there has been advances in the diagnosis and treatment of RA, there is a gap in knowledge regarding molecular and cellular mechanisms that cause RA. B cells contribute via their capacity to produce autoantibodies, proinflammatory cytokines and as antigen presenting cells. Early tolerance is altered in RA resulting in an increase in autoreactive B cells (3). The development of ACPA and rheumatoid factor autoantibodies precede disease development and expansion of ACPA and rheumatoid factor specific plasmablasts in RA patients with significant levels of somatic mutation, further indicating dysregulation in B cell maturation (4). Notably, B cell depletion with rituximab is efficacious in RA (5) and response to therapy with rituximab and abatacept (6) correlate with decreases in the level of ACPA. This highlights the need to better understand how B cells escape tolerance in RA and their role in propagating disease.

The role of IL-21 in B cell development and function has been well described. IL-21 induces B cell activation, expansion and the induction of plasma cells following stimulation and directly contributes to the generation of antibodies (7). IL-21 signals through the IL-21R and the common gamma chain (γc) and via the STAT signaling pathway. Phosphorylated STAT3 homodimerizes and translocates to the nucleus where it activates IL-21 target genes such as *PRDM1*, a critical transcription factor for plasma cell development (8, 9). It also has a direct role in the germinal center where it contributes to antibody production, including isotype switching and affinity maturation (10, 11).

Growing evidence suggests that IL-21 has a role in B cell dysfunction in RA. IL-21 levels in the serum and SF are increased in RA (12) and correlate to 28-joint count disease activity scores (13). A recent study demonstrated an increase in the frequency of CD19^+^IL-21R^+^ cells in RA compared to controls and observed enhanced proliferation and differentiation of B cells from RA patients compared to controls (13).

The importance of IL-21 in has also been demonstrated in murine models of rheumatoid arthritis. In the K/BxN model of spontaneous autoantibody mediated arthritis, K/BxN IL-21R^−/−^ mice are protected from initiation of arthritis or autoantibody development (14). Further, the treatment of K/BxN mice with an IL-21R-Fc fusion protein combined with an adjuvant delayed disease onset and decreased the severity of disease (14). The collagen-induced arthritis (CIA) IL-21R knockout murine model are resistant to CIA induction, demonstrate impaired antibody production and showed that IL-21 signaling in B cells was indispensable for the development of CIA (15). DBA/1 treated with bovine type II collagen mice were also used to investigate whether IL-21 contributed to disease development (16). Treating these mice with an IL-21R.Fc fusion protein reduced the clinical signs of collagen-induced arthritis, decreased antibody levels and decreased IL-6 levels thereby demonstrating a pathogenic role for IL-21 in mouse models of RA (16).

The goal of this study was to determine the mechanism that leads to increased IL-21R, extend our understanding of how it influences the function of RA B cells and more fully understand it in the context of disease. We demonstrate an increase in the expression of IL-21R on total and memory B cells in RA compared to controls, which positively correlated with pSTAT3 levels following IL-21 stimulation. Functionally, this increase in IL-21R on B cells was associated with increased expression of IL-21 target genes, plasmablast development and IL-6 production in response to IL-21. We link SP1 to the source of increased IL-21R based on increased SP1 expression in RA B cells and increased binding to the *IL21R* promoter region in RA. Together these findings suggest that increased expression of SP1 drives an increase in IL-21R, which potentiates the expansion of pathogenic B cells and autoantibody production in RA.

## Materials and methods

### Patients

**Figure 1:**
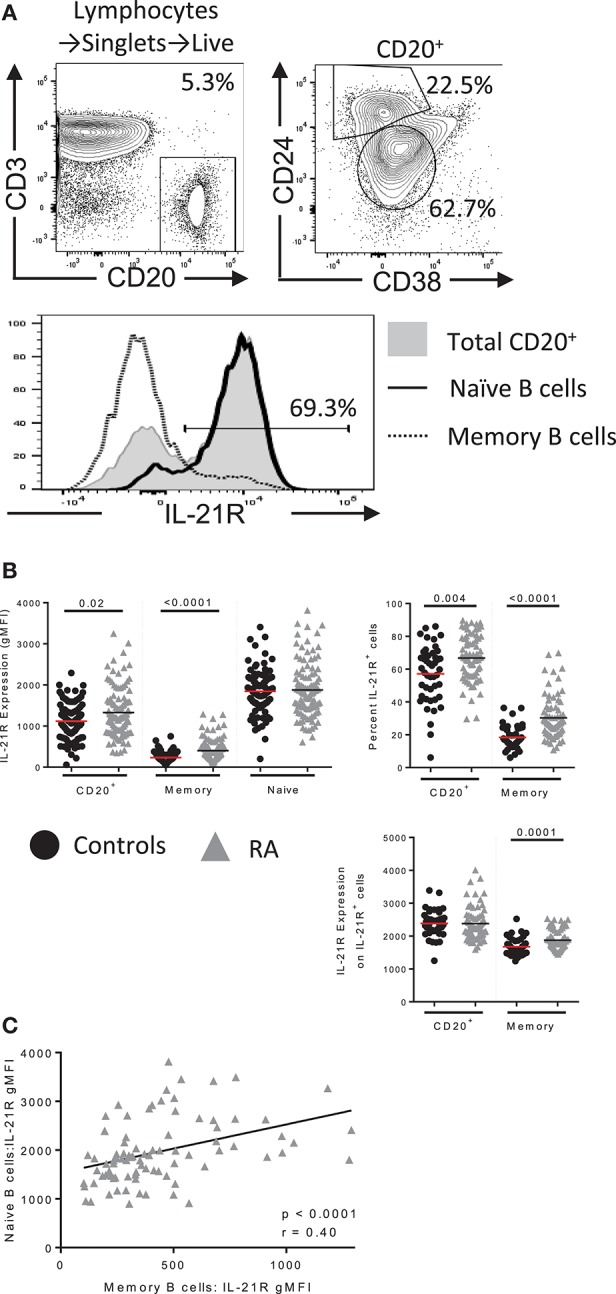
IL-21R expression is increased in B cells in RA. **(A)** (top) Gating scheme for total B, memory and naïve B cells with IL-21R expression in each population (bottom) **(B)** (left) IL-21R levels on CD20^+^, total memory (CD20^+^CD38^−^CD24^hi^) and naïve (CD20^+^CD38^int^CD24^int^) B cells in (black circles) healthy control (n = 46) and (gray triangles) RA subjects (n = 71) by flow cytometry. (Right) Percent of IL-21R^+^ cells (center, left) and IL-21R expression was quantified on IL-21R^+^ cells (bottom, right) in CD20^+^ and memory B cells in RA and control subjects. **(C)** IL-21R levels in memory and naïve B cells were correlated in RA subjects. Significance was determined using a Mann Whitney *U* test and a Pearson correlation.

All samples used in this study were from the Benaroya Research Institute Immune-Mediated Disease Registry and Repository. All patients gave written informed consent. Patient characteristics are summarized in Tables 1–4. RA subjects were drawn from a general rheumatology clinic and carry a diagnosis of RA based on the 2010 American College of Rheumatology criteria. There were two different cohorts of RA subjects. The first cohort (*N* = 110, Table 1) was cross-sectional with respect to disease duration, disease activity, antibody status and therapy although no one was on biologic DMARDs at the time of study. This cohort was compared to age-, gender-, and race-matched healthy control subjects (*N* = 93, Table 1). The second RA cohort (*N* = 52, Table 2) was selected to determine whether therapy had an effect on IL-21R or signaling responses. Individuals with SLE (*N* = 20, Table 3) carried a diagnosis of SLE based on the 1997 American College of Rheumatology criteria (17) and were age-, gender-, and race-matched to healthy control subjects (*N* = 21, Table 3). All individuals with MS had relapsing-remitting MS (*N* = 21, Table 4) based on the Revised McDonald Diagnostic Criteria for MS (18) and were age-, gender-, and race-matched to healthy control subjects (*N* = 27, Table 4). Healthy control subjects that were matched to the MS cohort are a subset of the healthy controls presented in Figure 1. Only samples that are matched are graphed together. Note all healthy control subjects had no history of autoimmune disease themselves or among their first-degree relatives. Disease status, gender, age, therapy and race was blinded until the conclusion of the study. All subjects were included in IL-21R expression studies, other assays were performed with selected subjects as defined in the figure legends. All PBMC samples were cryogenically frozen and thawed at the time of experiment except for synovial fluid/PBMC comparisons, which were fresh.

**Table 1:** RA and healthy control cohort characteristics.

	**RA subjects (*n* = 110)**	**Control subjects (*n* = 93)**
Age at draw (mean ± SD years)	51.7 ± 14.9	46.3 ± 14.2
Male/Female	24/86	25/68
Race	94.6% Caucasian,5.4% other	91.4% Caucasian, 8.6% other
Disease duration (mean ± SD years)	6.8 ± 8.4	
RAPID3 score (mean ± SD)	3.1 ± 2.2	
CCP^+^	60.9%	
RF^+^	57.3%	
Therapy	15% Steroid 50.9% DMARD 40% NSAID 10% Biologic	

*RAPID3, the routine assessment of patient index data with 3 measures (0–9); CCP, cyclic citrullinated peptide; RF, rheumatoid factor. Steroid therapy includes: prednisone (5–20 mg/day); DMARD includes: methotrexate (10–25 mg/week or 10 mg/2X weekly), sulfasalazine, hydroxychloroquine (200 mg/2X or 1X daily), azathioprine, and leflunomide; NSAID includes: ibuprofen, naproxen, aspirin, meloxicam, and celecoxib; and biologics includes: etanercept, abatacept, infliximab, and adalimumab*.

**Table 2:** Second RA cohort disease distribution.

	**RA subjects (*n* = 52)**
Age at draw (mean ± SD years)	57.3 ± 14.3
Male/Female	14/38
Race	92.3% Caucasian,7.7% other
Disease duration (mean ± SD years)	13.5 ± 10.8
RAPID3 score	2.6 ± 1.7
CCP^+^	86.5%
RF^+^	61.5%
Therapy	30% Steroid 75% DMARD 36.5% NSAID 55.8% Biologic

*RAPID3, the routine assessment of patient index data with 3 measures (0–9); CCP, cyclic citrullinated peptide; RF, rheumatoid factor. Steroid therapy includes: prednisone (5–20 mg/day); DMARD includes: methotrexate (10–25 mg/week or 10 mg/2X weekly), sulfasalazine, hydroxychloroquine (200 mg/2X or 1X daily), NSAID includes: ibuprofen, naproxen, aspirin, meloxicam, and celecoxib; and biologics includes: golimumab, etanercept, abatacept, infliximab, tofacitinib, and adalimumab*.

**Table 3:** SLE and healthy control cohort characteristics.

	**SLE subjects (*n* = 20)**	**Control subjects (*n* = 21)**
Age at draw (mean ± SD years)	45.0 ± 17.8	44.0 ± 16
Male/female	1/19	1/20
Race	60% Caucasian, 20% Asian, 10% African American, 10% Unknown	66.6% Caucasian, 19% Asian, 9.5% African American, 4.7% Pacific Islander
Disease duration (mean ± SD years)	10 ± 9.6	
SLEDAI score (mean ± SD)	4.6 ± 3.7	
ANA^+^	100%	
Therapy	45% Prednisone (2.5–7 mg/day) 80% Hydroxychloroquine	

*SLEDAI, Systemic lupus erythematosus disease activity index; ANA, antinuclear antibodies*.

**Table 4:** MS and healthy control cohort characteristics.

	**MS subjects (*n* = 21)**	**Control subjects (*n* = 27)**
Age at draw (mean ± SD years)	32.7 ± 8.6	33.9 ± 9.6
Male/female	8/13	13/14
Race	95% Caucasian, 4.7% Asian	88.9% Caucasian, 11.1% Asian
Disease duration (mean ± SD years)	4.4 ± 7.0	
Patient classifications	100% RRMS	
Therapy	100% untreated	

*RRMS, Relapsing remitting multiple sclerosis*.

### Synovial fluid processing

Synovial fluid was obtained from RA subjects undergoing therapeutic arthrocentesis. Synovial fluid samples were diluted 1:12 with 10% human serum RPMI 1640 (Gemini, GE). Diluted samples were treated with hyaluronidase (VWR) and benzonase (Sigma) for 30 minutes at 37°C, centrifuged and resuspended in 2 mL hemolytic buffer. Samples were quenched with 30 mL PBS, centrifuged, resuspended in 10% RPMI, filtered through a 100 μm cell strainer and washed with 10% RPMI media.

### Flow cytometry

PBMC were rested in XVIVO 15 (Lonza), stained with a viability dye (eBioscience) and blocked with Human TruStain FcX (Biolegend). PBMCs were incubated with CD19 (HIB19), CD20 (2H7), CD24 (ML5), CD10 (HI10a), IgM (MHM-88), CD3 (UCHT1), CD8 (RPA-T8), CD45RO (UCHL1), CD45RA (HI100), CD138 (MI15), IL-21R (17A12), from Biolegend; CD38 (HIT2), CD27 (L128), CD4 (SK3), CD27 (L128), Blimp-1 (5E7), γ_C_ (TUGh4), STAT3 (M59-50), from BD, SP1 (D4C3) from CST and IL-6 (MQ2-13AS) from eBioscience. IL-6 and IgM levels were determined after brefeldinA (Biolegend)/monensin (Biolegend) stimulation for 4 h, fixed with cytofix (BD), permeabilized with cytoperm (BD) followed by intracellular staining. Transcription factor staining was conducted according to the manufacturers protocol (BD). Where the mean fluorescent intensity (MFI) is analyzed we utilized the geometric mean fluorescent intensity. All flow cytometry experiments were acquired on a BD FACSCanto II (BD) and data were analyzed with FlowJo software (Tree Star).

### RNA flow cytometry

Intracellular RNA flow cytometry was conducted using the manufacturer's protocol (PrimeFlow RNA, Affymetrix, Santa Clara, CA). *RPL13A* RNA probe was used as a positive control and a no probe negative control was included. The following Type I RNA probes were obtained from eBioscience: *IL21R, SP1, PRDM1, SOCS1, SOCS3*, and miR155.

### Intracellular signaling

PBMCs were rested in XVIVO15 (Lonza). Cells were stimulated with media or IL-21 (50 ng/mL) (Miltenyi) for 45 min. PBMC was fixed with methanol and labeled with surface antibodies, intracellular CD20 antibody (H1, BD), and pSTAT3 (pY705) (4/P-STAT3, BD). Cells were stimulated with IL-10 (10 ng/mL) (BD) for 45 min or IL-4 (50 ng/mL) (R&D Systems) for 15 min. Cells stimulated with IL-10 were analyzed for pSTAT3 fold change levels and those stimulated with IL-4 were analyzed for pSTAT6 (pY641) (18/P-Stat6, BD). PSTAT3 fold change was calculated by the geometric mean of the stimulated well divided by the geometric mean of the unstimulated well.

### Gating B cells in flow cytometry

**Figure 2:**
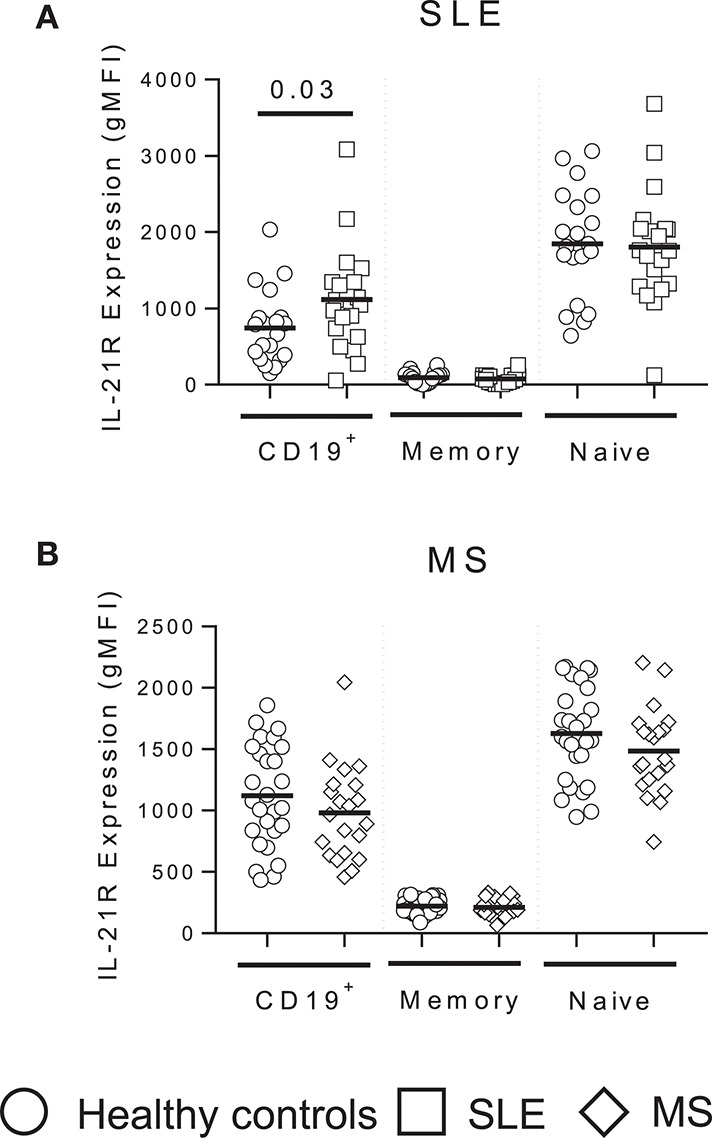
IL-21R expression is enhanced in total B cells in SLE subjects but unchanged in MS. IL-21R expression levels in SLE (white squares) **(A)** and MS (white triangles) **(B)** subjects compared to matched healthy controls (white circles) in total CD19^+^ B cells, total memory (CD20^+^CD38^−^CD24^hi^) and naïve (CD20^+^CD38^int^CD24^int^) B cells. Significance was determined using Mann Whitney *U* tests.

Example gating for B cell populations and IL-21R gating of a bimodal population is visualized in Figure 1A and is applied throughout the paper. CD20 was used to gate total B cells to allow a direct correlation in our signaling assays: the CD19 epitope is stripped during methanol permeabilization, thus we used surface CD20 to delineate B cells in Figure 1 and intracellular CD20 (clone: H1, BD) in Figure 2. For RNA flow cytometry, we used intracellular CD20 gating.

### B cells cultures and plasmablast induction

For 24 or 48 h cultures, PBMC was thawed and stimulated with IL-21 (50 ng/mL) (Miltenyi) with and without CD40L (5 μg/mL) (Peprotech). Four hours prior to harvest, brefeldinA/monensin were added to the cultures. Cells were harvested and stained for flow cytometry.

For plasmablast cultures, naïve B cells were isolated using the human naïve B cell isolation kit II (Miltenyi) and purity was assessed following 12-day cultures. Samples with purity >90% were plated at two million cells per well and cultured with IL-21 (15 pg/mL or 50 ng/mL) (Miltenyi) and CD40L (5 μg/mL) (Peprotech).

### Okadaic inhibitor assays

An aliquot of PBMC was removed to determine levels of SP1 and IL-21R prior to stimulation to obtain baseline levels. PBMC were plated with 0.1 μM Okadaic acid or DMSO control for 24 h.

### CCP3 IgG, rheumatoid factor IgM, IL-6 and IL-10 ELISAs

Serum from RA subjects was assessed for anti-cyclic citrullinated peptide 3 IgG (Inova Diagnostics), anti-rheumatoid factor IgM (Abcam), IL-6 (eBioscience), and IL-10 (eBioscience) levels according to the manufacturer's instructions.

### Chromatin immunoprecipitation

B cells were isolated by negative selection using the human B cell isolation kit II (Miltenyi). Cells were fixed with 4% paraformaldehyde for 10 minutes at 37°C. The cell pellet was frozen at −80°C until sample preparation. Magnetic beads were blocked (PBS/0.5% BSA) and incubated with either 10 μg SP1 antibody (07-645, Millipore) or control IgG overnight at 4°C. Nuclei were isolated the following day and sheared using a truChIP Chromatin Shearing Reagent Kit (Covaris), an AFA microTUBE (Covaris) and a M220 focused ultrasonicator (Covaris). Conditions for sonication: 20% duty cycle, 30 min. Sonicated samples were immunoprecipitated with magnetically labeled SP1 antibody or control IgG overnight at 4°C. The next day samples were reverse cross-linked. The Qiagen QIAquick PCR purification kit was used to purify the DNA.

### Quantitative RT-PCR

Gene expression analysis was performed using SYBR green real-time quantitative PCR on an ABI7900 HT analyzer (Applied Biosystems). Primers used to detect SP1 binding in the IL-21R promoter region were: forward (5′-GGTCCCTAAGAGGGAAGTGTCA−3′) and reverse (5′-GCACCCACTGTCACCAAAGG−3′) as previously published by Wu et al (19). Primers to detect SP1 binding outside of the *IL21R* gene are: site 1: forward (5′-GTCCCACAGGTCCCTAAGGT−3′) and reverse (5′-CCAGCAACTTCCCTGAGATG−3′); site 2: forward (5′-AAGGTAAGGCTGGGCTCAAT−3′) and reverse (5′-GCAATCTCAGGTCACTGCAA−3′).

### Statistics

Data was analyzed using GraphPad Prism. Statistical significance between controls and RA; or between RA-IL-21R^high^ compared to controls or IL-21R^low^, was assessed with a two-sided Mann Whitney *U* or Student's *t* test of which a *P* value of <0.05 was considered significantly different. A Mann Whitney *U* or Student's *t* test was selected after determining the normality of the groups with a D'Agostino-Pearson omnibus normality test. Correlations were assessed by calculating the Pearson's correlation coefficient, *r*.

The RA-IL-21R^high^ cohort was defined as subjects for whom the gMFI of IL-21R is two standard deviations (SD) above the mean of the control group. The RA-IL-21R^low^ cohort was defined by the IL-21R gMFI within two SD of the mean gMFI of the control group (see Figure 1 for further details). Samples selected from the RA-IL-21R^high^ and IL-21R^low^ groups were selected randomly once the groups were designated.

## Results

### IL-21R expression on B cells is enhanced in RA

Example gating for B cell populations and IL-21R gating of a bimodal population is visualized in Figure 1A and is applied throughout the paper. IL-21R expression was calculated on the entire B cell population as some subjects demonstrated a continuum of expression (Figures 1A,B). We observed increased IL-21R expression in total B cells as well as an increase in frequency of IL-21R^+^ B cells in RA subjects compared to healthy controls (Figure 1B). Frequency of IL-21R^+^ memory B cells was obtained by applying the total B cell IL-21R^+^ gate to the memory B cell population. This finding was most significant in the memory B cell population, however IL-21R gMFI in memory B cells correlated with IL-21R gMFI in naïve B cells indicating that this may extend to naïve B cells as well (Figure 1C). Also of note, IL-21R expression is decreased on memory compared to naïve B cells, this is due to the reduced need for IL-21 in mature B cells (20). No differences in IL-21R surface expression was present in T cells from RA subjects (Figure S1) indicating that this is a B cell specific finding.

B cells are increased in the RA joint, and have been shown to produce ACPA (21). To determine whether enhanced expression of IL-21R observed in the periphery is also seen in the RA joint we obtained paired synovial fluid and peripheral blood samples from RA subjects and measured IL-21R expression. Synovial fluid samples had an increased frequency of memory B cells with an increase in the frequency of switched memory as compared to peripheral blood (Figure S2A). IL-21R levels on synovial fluid B cells were as high as that seen in peripheral blood (Figures S2B–C). This suggests that IL-21 signaling in peripheral blood B cells may reflect that seen in the synovium.

Autoimmune disease immune phenotypes often demonstrate similarities suggesting similar mechanisms of action or disease pathogenesis. To determine whether IL-21R expression is a RA specific finding, we analyzed the IL-21R in SLE and MS subjects compared to screened healthy controls that were specifically matched for age, race and gender (Tables 2,3). We detected enhanced IL-21R expression in total CD19^+^ B cells in SLE subjects, though no differences were measured in memory or naïve B cell subsets (Figure 2A). Further, MS subjects demonstrated no differences in total CD19^+^, memory or naïve B cell subsets compared to controls (Figure 2B). This suggests that these findings are specific to autoimmune diseases under the rheumatic disease umbrella.

In our cohort of RA subjects with established disease, we observed only modest correlations with disease duration, ESR, rheumatoid factor IgM, and IL-21R expression, driven in part by a single outlier, and no correlations with CRP, CCP3-IgG or Rapid 3 score (Figure S3). We were able to assess IL-21R expression levels at two time points for a subset of RA subjects and found that it was stable over time (Figure S4). New therapies are under development to treat RA by targeting the IL-21 signaling pathway (22–25). We questioned whether current therapies used to treat RA may influence enhanced IL-21R expression that we have observed in some RA subjects. However, the increase in IL-21R on B cells was unrelated to therapeutic intervention (Table 2, Figure S5) and was modestly associated with disease duration. Together these data indicate that alterations in IL-21R expression we observe in RA are not likely to be secondary to either inflammation, longstanding RA and/or therapy.

### Comparison of IL-21R expression and response to IL-21

**Figure 3:**
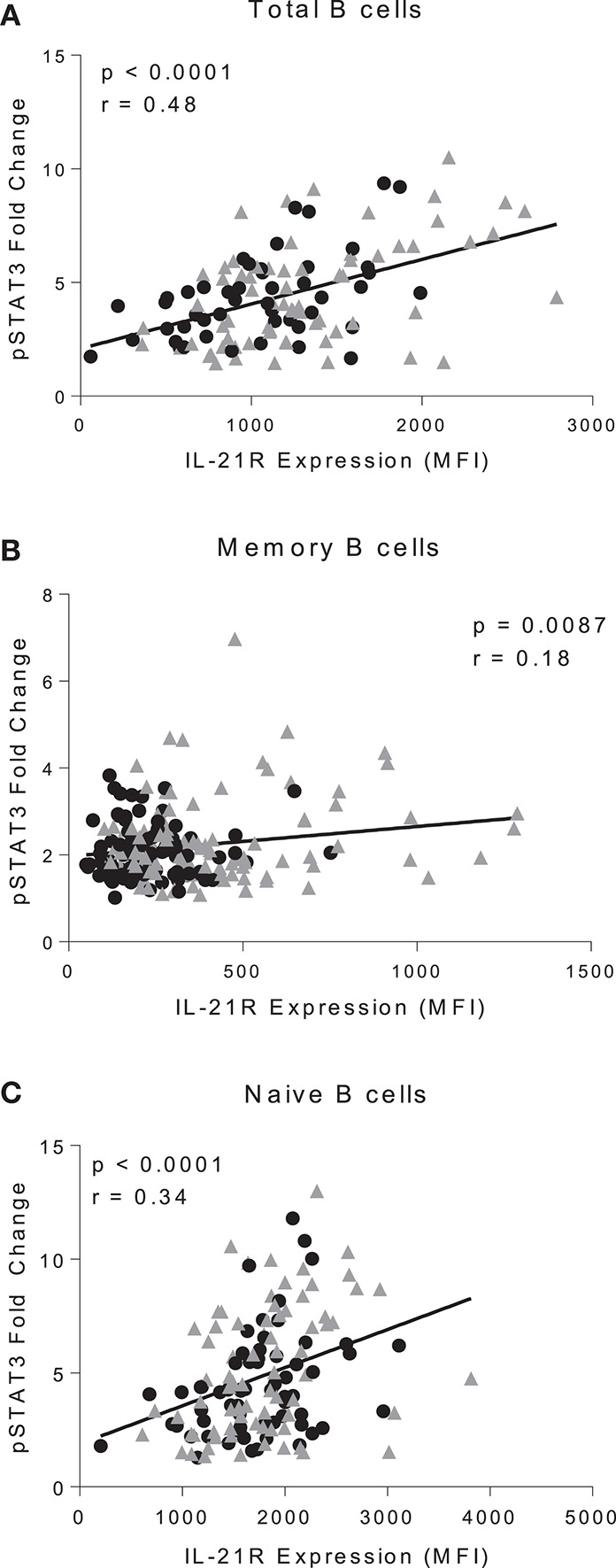
IL-21R levels correlate with IL-21-mediated pSTAT3 signaling. Correlation of IL-21R expression and pSTAT3 fold change in CD20^+^ **(A)**, total memory B cells **(B)**, and naïve B cells **(C)**. pSTAT3 levels were assessed by stimulating with IL-21 for 45 min and measuring pSTAT3 gMFI with no stimulation or with stimulation. Fold change was calculated by dividing IL-21 stimulation pSTAT3 gMFI by the no stimulation pSTAT3 gMFI. As there were no differences in slope between controls and RA patients, the correlation was conducted on both controls (black circles) and RA patients (gray triangles) (*n* = 116). Correlation was assessed with the Pearson correlation.

To determine whether increased IL-21R influences IL-21 signaling we measured pSTAT3 in PBMC stimulated with IL-21 (Figure S6A). pSTAT3 fold change was calculated by the geometric mean of stimulated cells divided by the geometric mean of unstimulated cells. We observed a significant correlation between IL-21R and pSTAT3 fold change in total (*p* < 0.0001), memory (*p* = 0.0087), and naïve (*p* < 0.0001) B cells (Figures 3A–C). There was no difference in γ_C_ expression or signaling through IL-4 (which signals through the γ_C_) suggesting that enhanced pSTAT3 response was not due to alterations in signaling via γ_C_ or a target downstream of it (Figures S6B,C). To determine whether all signaling through STAT3 was impacted, we examined baseline levels of STAT3 and pSTAT3 in response to IL-10, and found no significant differences between RA and controls (Figures S6D,E).

**Figure 4:**
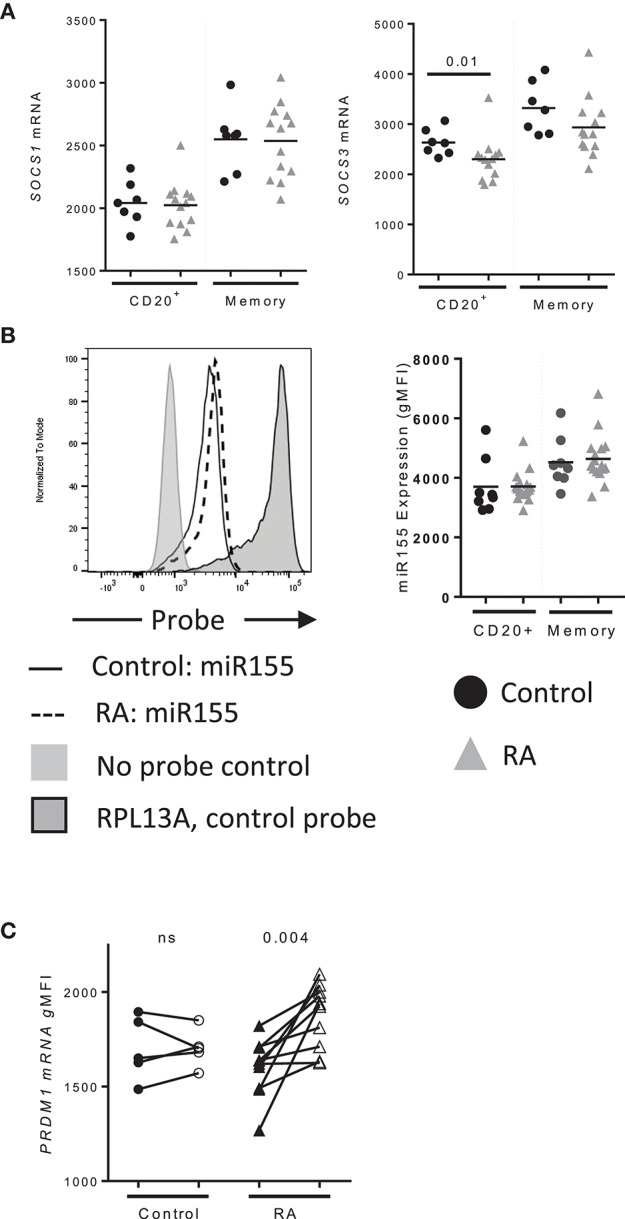
*PRDM1* levels are enhanced in memory B cells following IL-21 stimulation. **(A)** *SOCS1* (left) and *SOCS3* (right) mRNA expression in CD20^+^ and total memory B cells from (black circles) Control (*n* = 7), (gray triangles) RA (*n* = 14) subjects as assessed by mRNA flow cytometry. **(B)** miR155 levels in total and memory B cells (black circles) Control (*n* = 7), (gray triangles) RA (*n* = 14) subjects collected with miRNA flow cytometry. **(C)** *PRDM1* mRNA levels in memory B cells from control (circles, *n* = 5) and RA subjects (triangles, *n* = 10) at baseline (filled) and 48 h (unfilled) after stimulation with IL-21 (50 ng/mL) and measured by mRNA flow cytometry (*n* = 15). Significance was determined using Mann Whitney *U* tests.

To determine whether factors other than IL-21R play a role in IL-21 signaling in RA, we examined the expression of *SOCS1* and *SOCS3*, which negatively regulate IL-21 signaling (26, 27). We observed a significant decrease in *SOCS3* levels in B cells from RA compared to controls (Figure 4A). We did not detect a difference in *SOCS1* levels, nor did we observe a difference in miR155 levels in B cells between control and RA (Figure 4B). MiR155 acts by suppressing *SOCS1* and is elevated in B cells from RA that are ACPA^+^ (28). These data indicate IL-21R is the predominant factor implicated in the enhanced response to IL-21 in our RA cohort.

To determine whether IL-21 target genes in B cells were altered in RA, *PRDM1* levels were assessed following a stimulation with IL-21 (Figure 4C). Memory B cells from RA significantly induced *PRDM1* whereas there was no significant increase in *PRDM1* mRNA from controls (Figure 2D). These data suggest that increased IL-21R translates to increased IL-21 signaling resulting in enhancement of IL-21 target genes.

### Increased IL-21-mediated plasmablast differentiation is present in RA

**Figure 5:**
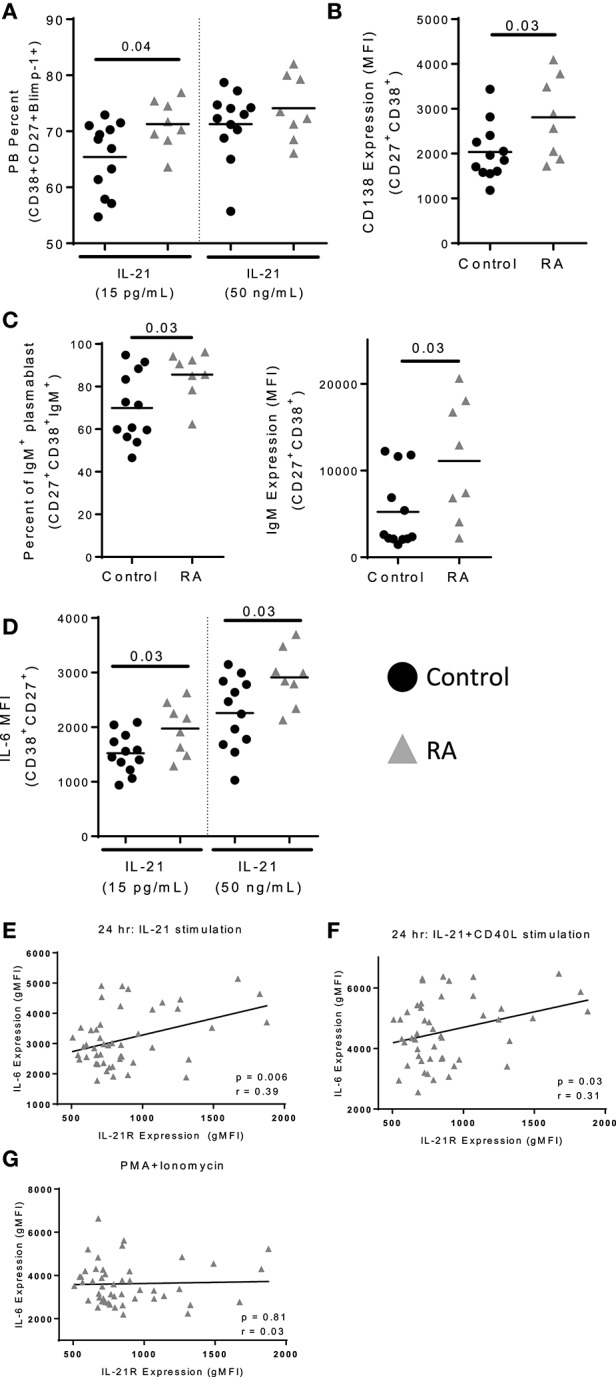
IL-21 mediated plasmablast differentiation is enhanced in RA. Naïve B cells were isolated from PBMC of control (*n* = 12) and RA patients (*n* = 8) and cultured with IL-21 (15 pg/mL or 50 ng/mL) and CD40L for 12 days. **(A)** Plasmablast percent was measured by staining for CD38^+^CD27^+^Blimp-1^+^ cells following 12 days of culture with either low (15 pg/ml) or high (50 ng/ml) levels of IL-21 in addition to CD40L. **(B)** CD138 expression was determined on plasmablasts (CD38^+^CD27^+^) from RA subjects and controls following high dose IL-21 and CD40L culture. **(C)** Frequency of IgM^+^ plasmablasts (CD38^+^CD27^+^) were assessed following 12 days of culture with high dose IL-21 and CD40L (left). IgM gMFI was assessed in plasmablasts by intracellular staining (right). **(D)** IL-6 gMFI was assessed on plasmablasts in response to low or high dose IL-21 in addition to CD40L stimulation in controls and RA subjects. **(E)** IL-21 expression correlates with IL-6 production following IL-21 stimulation **(F)** and IL-21+CD40L stimulation but not **(G)** PMA+Ionomycin stimulation. PBMCs from RA subjects (*n* = 52) were stimulated with IL-21 **(E)** or IL-21+CD40L **(F)** for 24 h. In the last 4 h of incubation, brefeldin A and monensin were added to prevent export of IL-6. The level of IL-6 was measured by flow cytometry on total B cells. IL-6 production was measured by flow cytometry. IL-21R expression was measured at day 0 on memory B cells. Black circles are controls and gray triangles are RA patients. Significance was assessed using Mann Whitney *U* tests. Correlation was assessed with the Pearson correlation.

IL-21 promotes the development of plasmablasts through the induction of *PRDM1*, which encodes Blimp-1, a transcription factor required for plasmablast development (29). To assess whether increased IL-21R expression enhances plasmablast development, we performed *in vitro* IL-21/CD40L stimulations, an approach successfully used by others to assess plasmablast differentiation (8, 30, 31). For these studies naïve B cells were used, as they would allow us to understand how enhanced IL-21 signaling influenced B cell development. The correlation between IL-21R on naïve and memory B cells among the subjects in our RA cohort support this approach. We observed a significant increase in the frequency of plasmablasts in RA as compared to control (Figure 5A), which was most pronounced at a low dose of IL-21. In addition, we observed an increase in expression of CD138 on RA plasmablasts (Figure 5B). The increase in frequency was among IgM^+^ plasmablasts (Figure 5C), with a corresponding increase in surface IgM levels (Figure 5C) though no difference in IgG levels (data not shown). Together, these data suggest increased plasmablast development in response to increased IL-21R expression.

### IL-21R expression and IL-6 production are correlated in B cells

Plasmablasts produce increased quantities of IL-6 as compared to naïve B cells (32). We observed an increase in IL-6 production by the RA plasmablasts in *in vitro* plasmablast cultures compared to controls (Figure 5D). Since IL-6 production was altered in cultures, we investigated the relationship between IL-21R and IL-6 production. In RA, we found that IL-21R on memory B cells correlated with IL-6 production following stimulation with IL-21 or IL-21+CD40L (Figures 5E–F). In contrast, there was no correlation between IL-21R and IL-6 production or intracellular IL-6 levels following stimulation with PMA/ionomycin (Figure 5G, Figure S7A), suggesting that the differences in IL-6 production are dependent on IL-21. Although not significant, in our cohort there was a trend suggesting a correlation between IL-21R and increased serum IL-6 levels in RA (*p* = 0.07) (Figures S7B,C). In addition, no correlation with levels of IL-10 was seen in these subjects (Figures S7D,E). Together, this suggests that IL-6 production by B cells is IL-21 signaling dependent.

### SP1 binding to the IL-21R promoter

**Figure 6:**
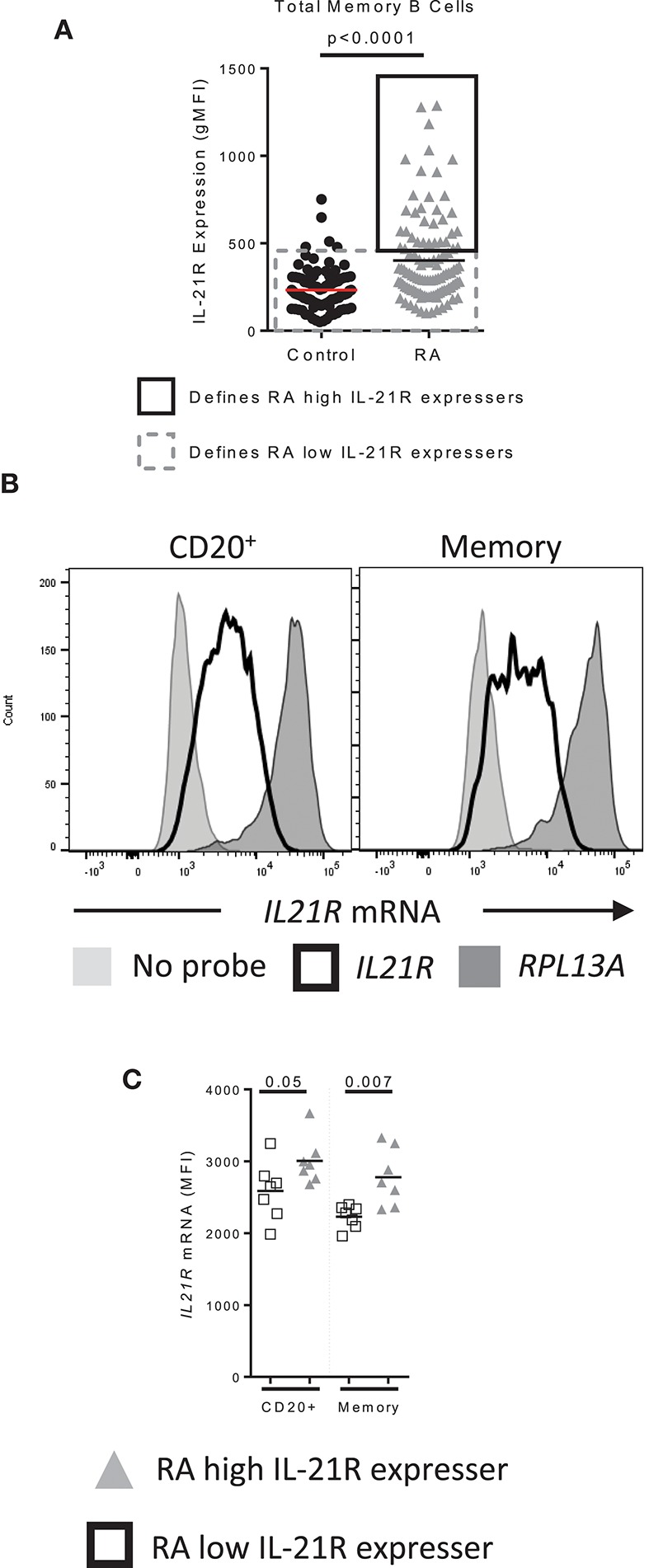
IL-21R transcript levels are increased in RA subjects with high levels of IL-21R **(A)** Data from Figure 1A used to show how RA high IL-21R expressers and RA low IL-21R expressers are defined. RA high IL-21R expressers in the solid black box express IL-21R at levels two times the standard deviation of the mean of the control samples. RA low IL-21R expressers in the gray dashed box are less than two times the standard deviation of the mean of the control samples. **(B)** Representation of the positive control probe (*RPL13A)* (dark gray histogram), *IL21R* (black line histogram) and negative control (no probe) (light gray histogram) in CD20^+^ or memory B cells in a control subject. **(C)** *IL21R* mRNA levels in total and memory B cells from RA high IL-21R expressers (gray triangles, *n* = 7) and RA low IL-21R expressers (white squares, *n* = 7) Significance was determined using a Mann Whitney *U* test.

We next asked whether increased IL-21R levels were due to increased transcription. RNA flow was used for this purpose as it can be used to determine protein and mRNA levels simultaneously in rare cell types (representative staining, Figure S8A). Subjects were randomly selected from RA high IL-21R (RA-IL-21R^high^) and RA low IL-21R (RA-IL-21R^low^) groups. We defined RA-IL-21R^high^ as subjects for whom the gMFI of IL-21R is two standard deviations (SD) above the mean of the control group. RA-IL-21R^low^ were RA subjects with IL-21R gMFI within two SD of the mean gMFI of the control group (Figure 6A). *IL21R* mRNA levels were significantly increased in RA-IL-21R^high^ expressers compared to RA-IL-21R^low^ in total and memory B cells (Figures 6B,C) suggesting that enhanced IL-21R on RA B cells is transcriptionally regulated.

**Figure 7:**
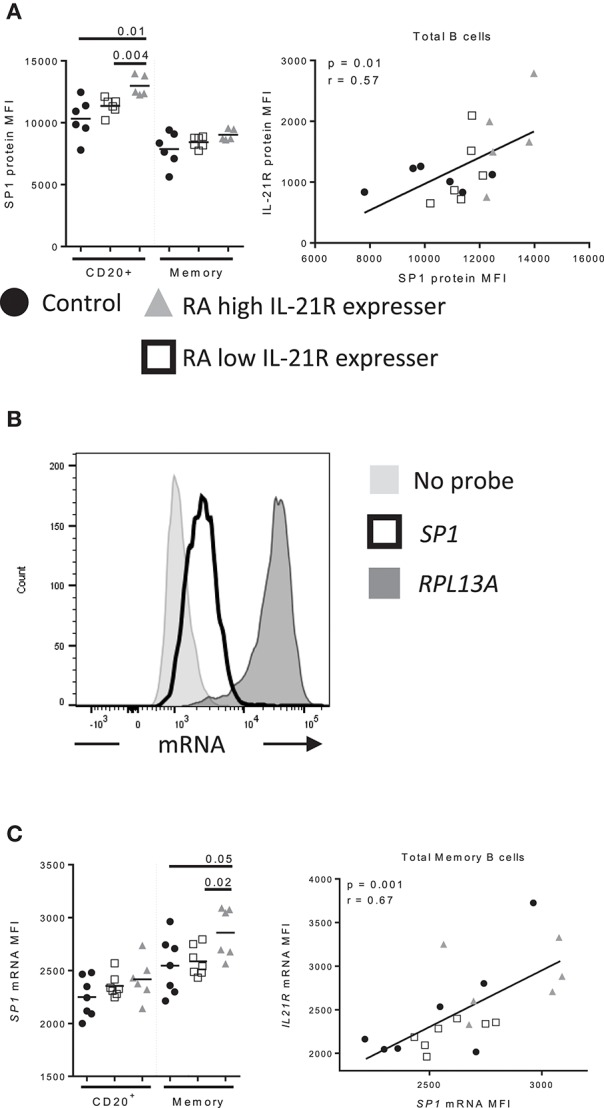
Increased SP1 expression correlates with increased IL-21R expression in memory B cells in RA subjects **(A)** SP1 protein levels were assessed by flow cytometry in control (*n* = 6, black circles), RA low IL-21R expressers (*n* = 6; open squares) and RA high IL-21R expressers (*n* = 5; gray triangles) in CD20^+^ and memory B cells (CD20^+^CD38^−^CD24^+^) (left). SP1 protein levels from left were correlated with IL-21R protein expression in total B cells in RA and control subjects (*n* = 17) (right). **(B)** Representative histogram of mRNA levels from a no probe negative control, *SP1* and positive control probe, *RPL13A*. **(C)** (left) *SP1* mRNA levels were determined in total memory B cells in controls (*n* = 7, black circles), RA low IL-21R expressers (*n* = 7; open squares) and RA high IL-21R expressers (*n* = 6; gray triangles). (right) *SP1* mRNA levels from left were correlated with IL-21R protein expression in memory B cells in RA and control subjects combined (*n* = 20). Significance was determined using Mann Whitney U tests (to compare RA-IL-21^high^ to controls and RA-IL-21^low^) and correlations were assessed with Pearson correlations.

Specificity protein 1 (SP1) has been reported to regulate IL-21R expression in T cells (19). We assessed whether SP1 is responsible for enhanced IL-21R in B cells in RA and we found that SP1 levels were increased in B cells from RA-IL-21R^high^ compared to controls and RA-IL-21R^low^ subjects (Figure 7A). In addition, SP1 protein levels correlated significantly with IL-21R protein levels in B cells (Figure 7A). *SP1* mRNA levels were increased in memory B cells from RA-IL-21R^high^ expressers (Figures 7B,C) and this significantly correlated with *IL21R* mRNA levels (Figure 7C).

**Figure 8:**
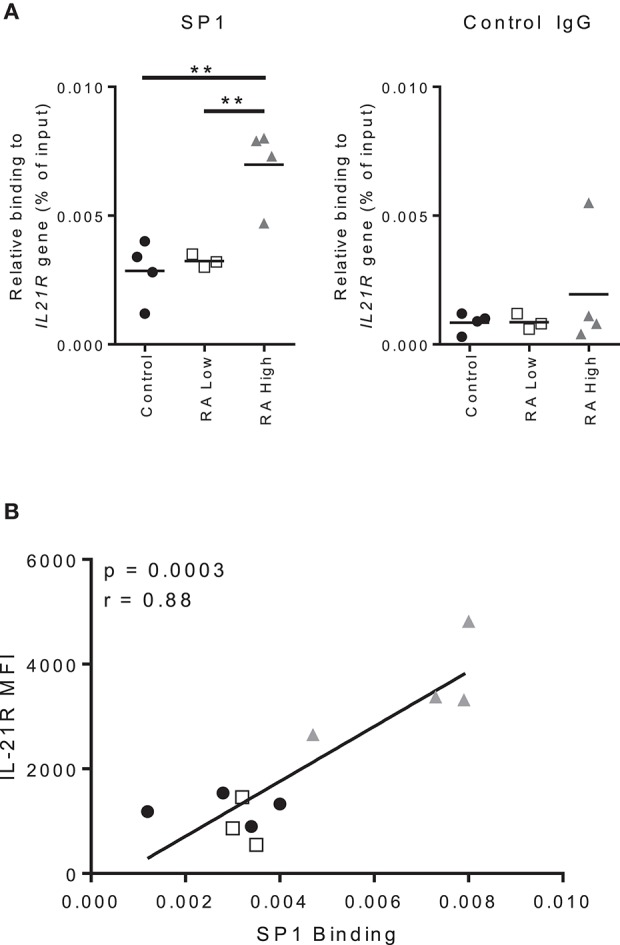
Increased IL-21R expression correlates with increased SP1 binding to the IL-21R promoter in RA. **(A)** Total B cells were isolated from whole blood from RA patients and controls. SP1 binding to the IL21R promoter region and negative control IgG binding were determined using ChIP-qPCR analysis. The results are presented relative to input DNA. Significance was assessed using the student's *t*-test; *n* = 4 [Control (black circle), RA High (gray triangle)] *n* = 3 [RA Low (white square)]. ^**^p < 0.01. **(B)** Correlation between IL-21R expression in total B cells as assessed by flow cytometry and SP1 relative binding to the IL21R gene as assessed by ChIP-qPCR and presented in A. One subject from the RA low cohort was excluded due to low purity of the B cell population. Significance was determined using Mann Whitney U tests **(A)** and Pearson correlations **(B)**.

We next assessed the binding of SP1 to the *IL21R* promoter in B cells. As before, we analyzed B cells from controls, RA-IL-21R^high^ and IL-21R^low^ subjects and performed ChIP-qPCR on purified B cells; we analyzed the SP1 binding site that were identified in the *IL21R* promoter in T cells (19). We used total B cells as we could not obtain sufficient quantities of memory B cells from RA and we observed alterations in total B cells, as well as memory B cells, in IL-21R and SP1 expression. We observed enhanced binding of SP1 to the *IL21R* promoter in RA-IL-21R^high^ B cells compared to both controls and RA-IL-21^low^ subjects (Figure 8A). There were no significant differences in SP1 binding when we analyzed sites outside the *IL21R* promoter region (Figure S8). Importantly, there was a strong and significant correlation between IL-21R and SP1 binding to the *IL21R* promoter (Figure 8B).

To explore this mechanism further, we used okadaic acid to determine whether inhibition of SP1 dephosphorylation would result in a decrease in IL-21R on B cells. Okadaic acid specifically blocks protein phosphatase 2A (PP2A) which has been shown to control the dephosphorylation of SP1 (33). Dephosphorylation of SP1 has been previously reported to be required for SP1 induced IL-21R in T cells (19). We observed a significant reduction in the levels of protein for both SP1 and IL-21R in controls, RA-IL-21R^low^ and IL-21R^high^ subjects following okadaic acid treatment which was normalized to the DMSO control (Figure S9). These findings suggest that enhanced binding of SP1 to the *IL21R* promoter may be responsible for increased IL-21R expression in B cells in RA.

## Discussion

We find that RA subjects with elevated IL-21R expression demonstrate hyper-responsiveness to IL-21, increased plasmablast differentiation and IL-6 production. These features could participate in early RA by promoting the development of autoantibodies and the persistent inflammation in late disease via the production of IL-6. These findings are consistent with the observation that circulating plasmablasts are increased in the setting of active RA disease and supports a role for the IL-21/IL-21R pathway in autoantibody formation in RA (34). A role for IL-21 signaling in autoantibody production is supported by prior work showing that serum IL-21 has a significant, albeit modest, correlation with CCP antibodies and a trend suggesting correlation with rheumatoid factor IgG antibodies in a previous study (13). Similarly, we found a modest correlation between IL-21R on RA memory B cells and levels of rheumatoid factor IgM in our study.

The correlation between memory and naïve B cell IL-21R expression in RA subjects suggests that IL-21R expression is typically downregulated during development/activation (20). In our study, IL-21R expression is maintained as naïve B cells progress to memory B cells, which appears to result in enhanced plasma cell differentiation. Enhanced IL-21 expression has also been shown to participate in the generation of autoreactive CD11c^hi^t-bet^+^ B cells as described in human SLE (35). Interestingly, these cells can be induced by IL-21 (36, 37) or IFN-γ (36, 38), and are present in healthy control subjects (39). Other studies have shown that CD11c^hi^t-bet^+^ B cells are expanded in SLE (35, 40) and MS (41). These cells have also been characterized in RA by their low expression of CD21 where we previously demonstrated an increased frequency of these cells (39). Given the involvement of IL-21 in the development of CD11c^hi^t-bet^+^ B cells, our study suggests that the enhanced IL-21R we observe may contribute to the development of these autoreactive cells. Further investigation is needed to determine how enhanced IL-21R signaling contributes to the development of these cells in RA.

Multiple factors may influence the enhanced plasmablast development and IL-6 production by B cells in RA patients. Our group previously observed an increase of approximately 2% in unconventional memory cells (CD19^+^CD27^−^CD21^−^) in RA subjects compared to controls in the naïve B cell population (39). Unconventional memory cells have the capacity to rapidly mature into plasma cells (42). The frequency of unconventional memory cells in the maturation cultures, while not zero, is likely too small to make an impact. Further, *in vivo*, increased IL-6 production in response to IL-21, by both memory B cells and plasmablasts, may be a driving factor in the expansion of T follicular helper and Th17 cells in RA and ultimately promote B cell maturation and autoantibody production (12, 13, 32, 43, 44).

SLE subjects demonstrated enhanced IL-21R expression on total CD19^+^ B cells compared to controls while MS subjects did not. This finding was modest, and not observed in memory or naïve B cell subsets. Notably, there are several papers in the literature that have assessed IL-21R expression in SLE subjects. These papers provide opposing views to whether SLE subjects express increased, decreased or no difference in expression compared to controls (45–47). Le Coz et al. only observed an increase on memory B cells in SLE subjects compared to controls (46). SLE is a heterogeneous disease and our cohort was drawn from our clinic population which was not controlled for disease activity at time of draw, disease duration or organ involvement. The analysis of SLE subjects is further complicated by the involvement of the *IL21R* SNP rs3093301 in SLE (48, 49), which is not associated with RA or MS. It is interesting to note that that about 44% of RA subjects will develop at least one SLE criteria in addition to arthritis (50). This suggests there may be similar pathogenic mechanisms between diseases (50) and our findings presented here suggest that the mechanisms of IL-21R regulation may be shared between SLE and RA.

In this study we determined the mechanism that leads to enhanced IL-21R in RA, show that IL-21R mRNA levels are increased in RA B cells and investigated the role of SP1 in the regulation of IL-21R expression. Augmented transcriptional activity of SP1 has been described in fibroblast-like synovial cells and in bone marrow mononuclear cells in RA (51–53). T cell receptor induced SP1 activity has been shown to mediate IL-21R expression in human T cells (19). Our finding that SP1 regulates the maintenance of baseline IL-21R in human B cells in RA is, to our knowledge, a novel insight as the role of SP1 in induction of IL-21R in B cells and its role in modulating IL-21R expression in RA has not been investigated. Chronic inflammation experienced by RA subjects may drive SP1 and IL-21R expression, promoting IL-6 production and the expansion of the autoantigen specific B cells ultimately contributing to the expanding specificities of ACPA and the maturation of high affinity responses seen in RA over time. Alternatively, genetic or epigenetic factors may lead to SP1 driven IL-21R expression early in disease in the absence of inflammation. In this study, the role of IL-21R expression in the development of RA was not investigated. In this setting, increased IL-21R expression on B cells could play a role in the development of autoantibodies, epitope spreading and the increased inflammatory mediators (54) seen in progression to disease.

Other mechanisms may contribute to increased IL-21R expression or IL-21 responsiveness in the setting of RA. We observed a significant decrease in *SOCS3* in B cells in RA. As *SOCS3* is a negative regulator of IL-21 signaling, it may contribute to the enhanced response to IL-21 seen in RA B cells. Prior studies have found *SOCS1* and *SOCS3* transcripts in PBMC to be increased in RA (55). Due to the relatively small percentage of B cells in PBMC, it is unlikely that the *SOCS1* and *SOCS3* expression in B cells would be reflected in this analysis. No differences in miR155 expression between RA and controls in our cohort were observed which is in contrast to a report from Alivernini's group that miR155 levels are elevated in B cells in RA (28). These differences may be due to cohort characteristics and/or the method of miR155 detection; we used miRNA flow cytometry whereas Alivernini's group used qPCR. In either case, a decrease in *SOCS3* expression in RA could contribute to enhanced IL-21/IL-21R signaling.

Defining the role of IL-21/IL-21R signaling in RA is important because of the interest in blocking this pathway therapeutically by targeting IL-21, IL-21R (56) and the Janus kinases (JAK) (57, 58). Two JAK inhibitors are approved for patients with RA (Tofacitinib and Baricitinib) and both demonstrate efficacy as defined by improvements in DAS28-CRP scores (59–62); however, these therapies influence multiple cytokine signaling pathways, increasing the importance of understanding the impact of alterations in the IL21/IL-21R signaling pathway on disease development and progression. Studies as we present here could shed light on patients most likely to respond to IL-21 targeted therapies, and the ideal time point for intervention. Our findings in a cross-sectional cohort of individuals with RA and controls suggest that IL-21 based therapies for RA may be more efficacious if targeted to individuals with increased IL-21R expression on memory B cells; studies that evaluate response to therapy, requiring a longitudinal cohort with samples collected before and after initiation of therapy would be of particular interest.

We have shown that IL-21R expression is increased on total and memory B cells in peripheral blood and synovial fluid from RA subjects. Increased IL-21R in peripheral blood correlates with enhanced response to IL-21 with respect to *PRDM1* expression, plasmablast differentiation and IL-6 production. Mechanistically we showed that the increased IL-21R was due to enhanced binding of the transcription factor, SP1. Together, these studies highlight the importance of IL-21 signaling in B cells and provide insight into how to target therapies to the IL-21 signaling pathway in RA.

## Ethics statement

All subjects gave written informed consent in accordance with the Declaration of Helsinki and according to the Institutional Review Board-approved protocols at both the Benaroya Research Institute, and the VA Puget Sound Health Care System.

## Author contributions

ED designed the research studies, conducted experiments, acquired data, analyzed data, and wrote the manuscript. AM conducted experiments, acquired data, and analyzed data. AH wrote the manuscript. JC and BN recruited subjects. JB recruited subjects, designed the research studies and wrote the manuscript.

### Conflict of interest statement

The authors declare that the research was conducted in the absence of any commercial or financial relationships that could be construed as a potential conflict of interest.
