# Corrigendum: Increased Binding of Specificity Protein 1 to the *IL21R* Promoter in B Cells Results in Enhanced B Cell Responses in Rheumatoid Arthritis

**DOI:** 10.3389/fimmu.2019.01122

**Published:** 2019-05-16

**Authors:** Elizabeth M. Dam, Alison C. Maier, Anne M. Hocking, Jeffrey Carlin, Bernard Ng, Jane H. Buckner

**Affiliations:** ^1^Translational Research Program, Benaroya Research Institute, Seattle, WA, United States; ^2^Division of Rheumatology, Virginia Mason Medical Center, Seattle, WA, United States; ^3^Rheumatology Section, VA Puget Sound Health Care System, Seattle, WA, United States; ^4^Division of Rheumatology, Department of Medicine, University of Washington, Seattle, WA, United States

**Keywords:** rheumatoid arthritis, B cells, IL-21R, specificity protein 1, IL-6

Bernard Ng was not included as an author in the published article. The corrected Author Contributions Statement appears below.

“ED designed the research studies, conducted experiments, acquired data, analyzed data, and wrote the manuscript. AM conducted experiments, acquired data, and analyzed data. AH wrote the manuscript. JC and BN recruited subjects. JB recruited subjects, designed the research studies and wrote the manuscript.”

Furthermore, in the original article, there was an error. The Material and Methods section incorrectly stated that: “All samples used in this study were from participants in the Benaroya Research Institute Immune-Mediated Disease Registry and Repository.” Although the samples from Veterans recruited by Dr. Bernard Ng at the VA Puget Sound Health Care System were deposited into the Benaroya Research Institute Immune-Mediated Disease Registry and Repository, the Veterans were not participants in the registry.

A correction has been made to the ***Materials and Methods***, subsection ***Patients***, paragraph one*:*

“All samples used in this study were from the Benaroya Research Institute Immune-Mediated Disease Registry and Repository. All patients gave written informed consent. Patient characteristics are summarized in Tables 1–4. RA subjects were drawn from a general rheumatology clinic and carry a diagnosis of RA based on the 2010 American College of Rheumatology criteria. There were two different cohorts of RA subjects. The first cohort (*N* = 110, Table 1) was cross-sectional with respect to disease duration, disease activity, antibody status and therapy although no one was on biologic DMARDs at the time of study. This cohort was compared to age-, gender-, and race-matched healthy control subjects (*N* = 93, Table 1). The second RA cohort (*N* = 52, Table 2) was selected to determine whether therapy had an effect on IL-21R or signaling responses. Individuals with SLE (*N* = 20, Table 3) carried a diagnosis of SLE based on the 1997 American College of Rheumatology criteria (17) and were age-, gender-, and race-matched to healthy control subjects (*N* = 21, Table 3). All individuals with MS had relapsing-remitting MS (*N* = 21, Table 4) based on the Revised McDonald Diagnostic Criteria for MS (18) and were age-, gender-, and race-matched to healthy control subjects (*N* = 27, Table 4). Healthy control subjects that were matched to the MS cohort are a subset of the healthy controls presented in Figure 1. Only samples that are matched are graphed together. Note all healthy control subjects had no history of autoimmune disease themselves or among their first-degree relatives. Disease status, gender, age, therapy and race was blinded until the conclusion of the study. All subjects were included in IL-21R expression studies, other assays were performed with selected subjects as defined in the figure legends. All PBMC samples were cryogenically frozen and thawed at the time of experiment except for synovial fluid/PBMC comparisons, which were fresh.”

Additionally, the Ethics Statement did not include the Institutional Review Board at the VA Puget Sound Health Care System.

A correction has thus been made to the ***Ethics Statement****:*

“All subjects gave written informed consent in accordance with the Declaration of Helsinki and according to the Institutional Review Board-approved protocols at both the Benaroya Research Institute, and the VA Puget Sound Health Care System.”

The authors apologize for these errors and state that they do not change the scientific conclusions of the article in any way. The original article has been updated.

