# Supplementary material for: Increased Binding of Specificity Protein 1 to the IL21R Promoter in B Cells Results in Enhanced B Cell Responses in Rheumatoid Arthritis
Source: Front Immunol. 2018 Sep 5;9:1978. doi: 10.3389/fimmu.2018.01978 (PMC6134023; doi:10.3389/fimmu.2018.01978)
Supplement: Supplementary file 1 [file Image_1.pdf]

Supplemental Figures

Increased binding of specificity protein 1 to the *IL21R* promoter in B cells results in enhanced B cell responses in rheumatoid arthritis

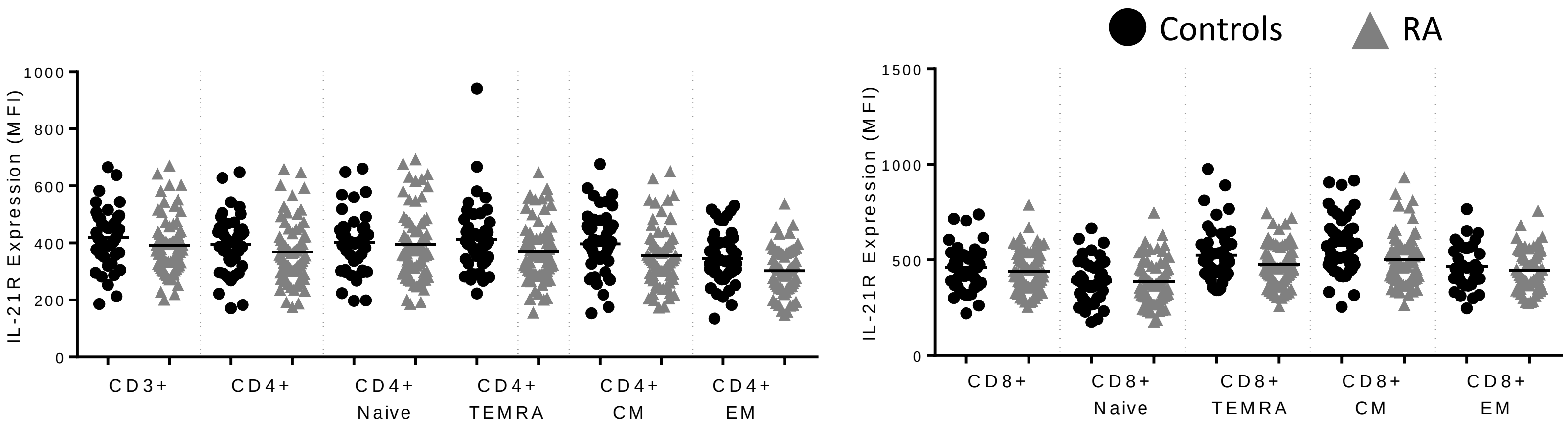

Supplemental Figure 1: No change in IL-21 receptor expression in T cells in RA patients compared to controls. (A) IL-21R expression was measured in $CD4^+$ (left) and $CD8^+$ (right) T cell subsets in controls compared to RA patients (●) Controls (n = 46) and (Δ) RA (n = 71). After gating $CD3^+$ cells, $CD4^+$ and $CD8^+$ cells were positively gated followed by naïve ($CD45RA^+CD27^+$), TEMRA ($CD45RA^+CD27^-$), central memory (CM, $CD45RO^+CD27^+$) and effector memory (EM, $CD45RO^+CD27^-$). Significance was assessed using a Mann Whitney U test.

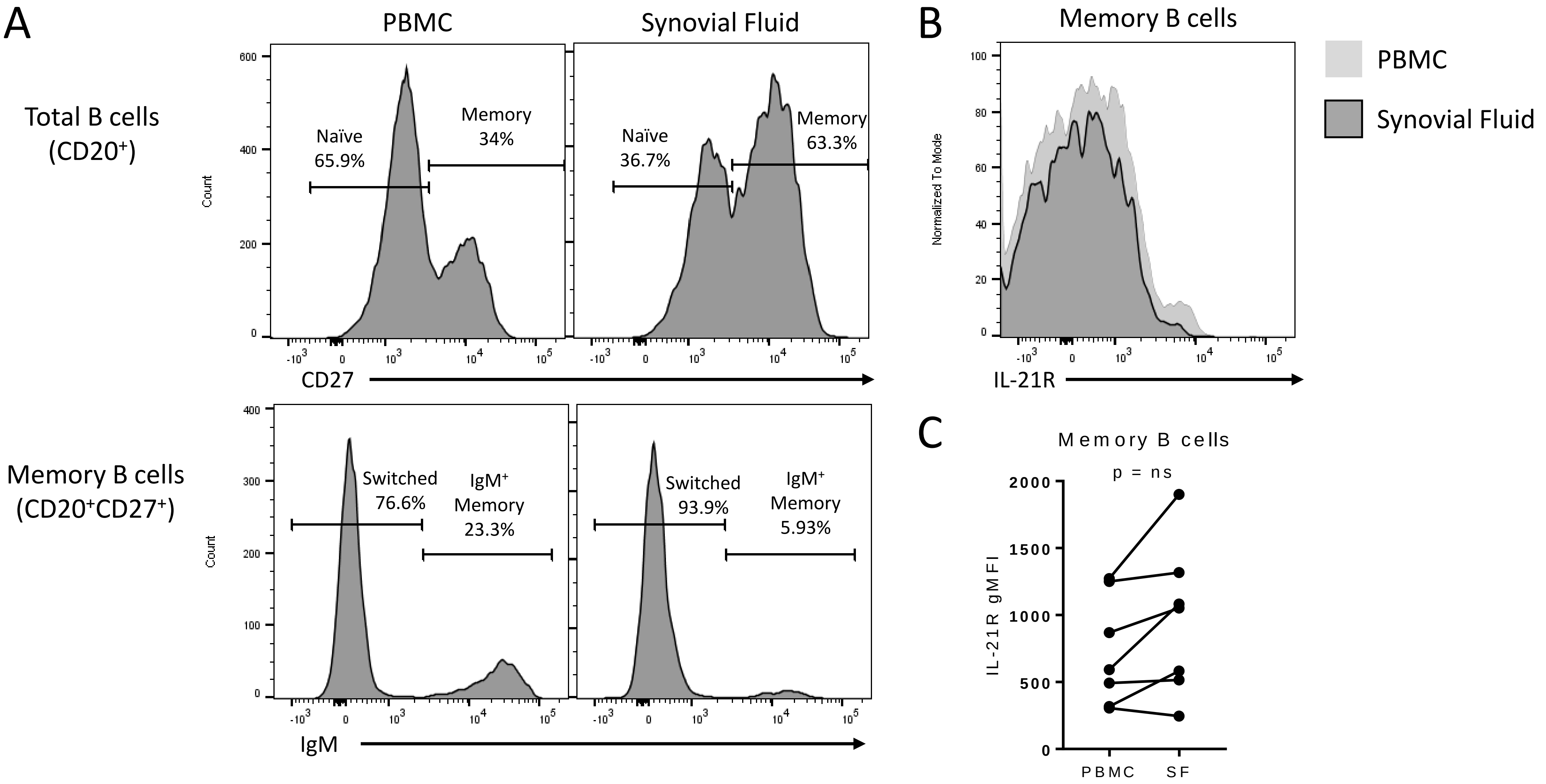

Supplemental Figure 2: IL-21R expression in memory B cells in the synovial fluid is the equivalent to those in the periphery in RA patients. (A) Cells from peripheral blood (PBMC) and synovial fluid were analyzed by staining for total B cells ($CD20^{+}$) followed by gating for naïve ($CD27^{-}$) and memory ($CD27^{+}$) B cell subsets (top). Memory B cells were assessed for IgM^{+} and switched memory populations (IgM^{-}) (bottom). **(B)** Representative IL-21R staining of total memory B cells from PBMC and synovial fluid. **(C)** Comparison of IL-21R expression levels on memory B cells from PBMC and synovial fluid (SF) from the same RA subject. In C, a paired t-test was used to assess significance between PBMC and SF of the same subject (N = 7).

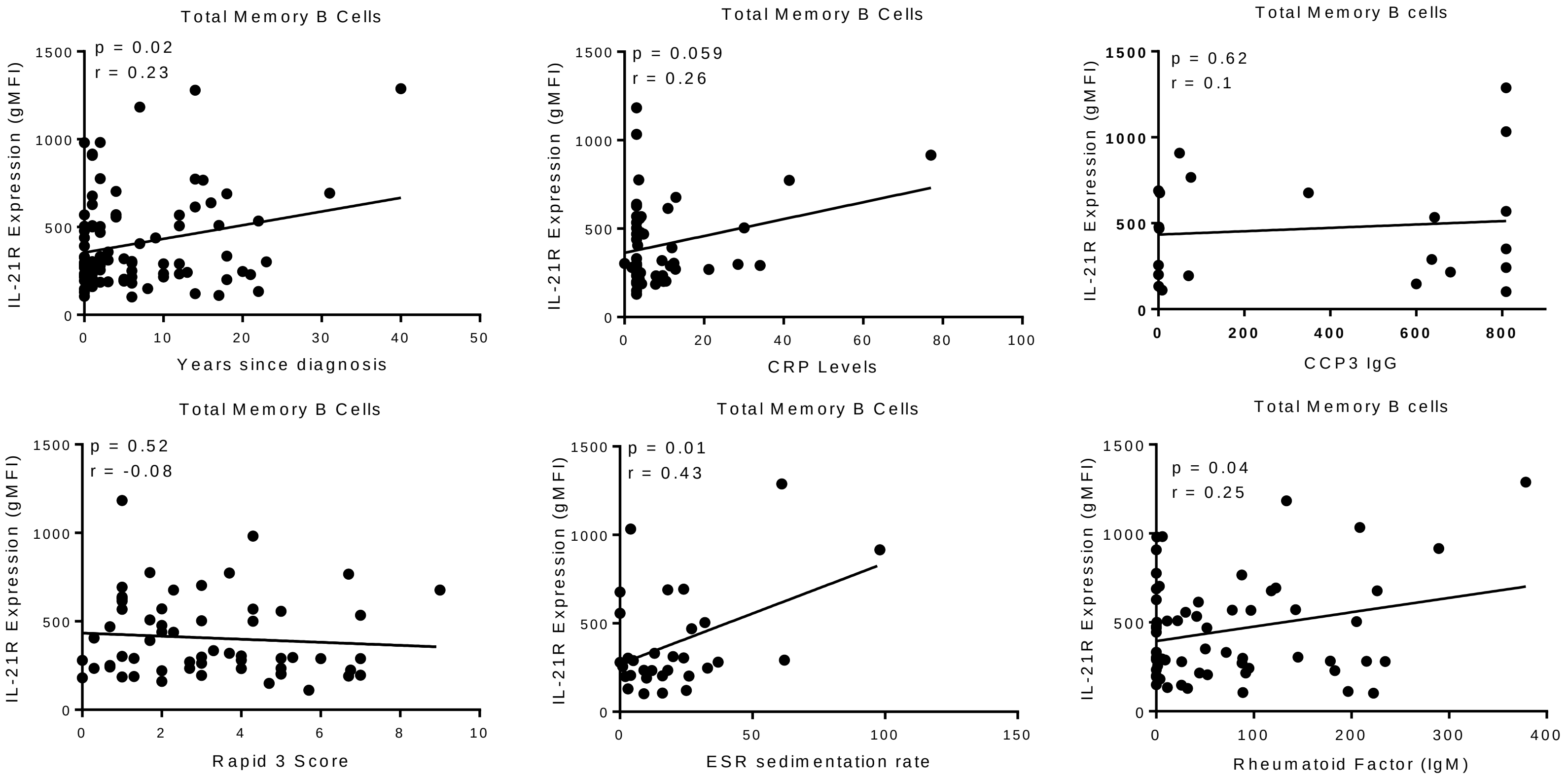

Supplemental Figure 3: Correlation between IL-21R expression, years since diagnosis and ESR sedimentation rate. IL-21R expression in total memory B cells obtained as described in (Figure 1A) was correlated to years since diagnosis (n = 96) (upper left), rapid 3 score (n = 59) (lower left), CRP levels (n = 50) (upper middle), ESR sedimentation rate (n = 32) (lower middle), CCP3 IgG (n = 22) (upper right) and rheumatoid factor IgM (n = 62) (lower right) in RA patients. Significant correlation was assessed using Pearson correlation where r is the correlation coefficient.

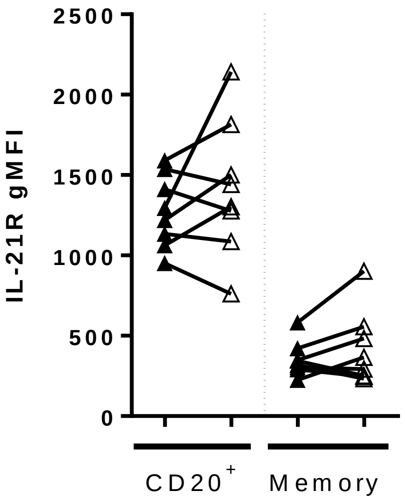

Supplemental Figure 4: IL-12R expression is stable. IL-12R expression was assessed in RA subjects at two time points. The two timepoints were between 2-5 years apart.

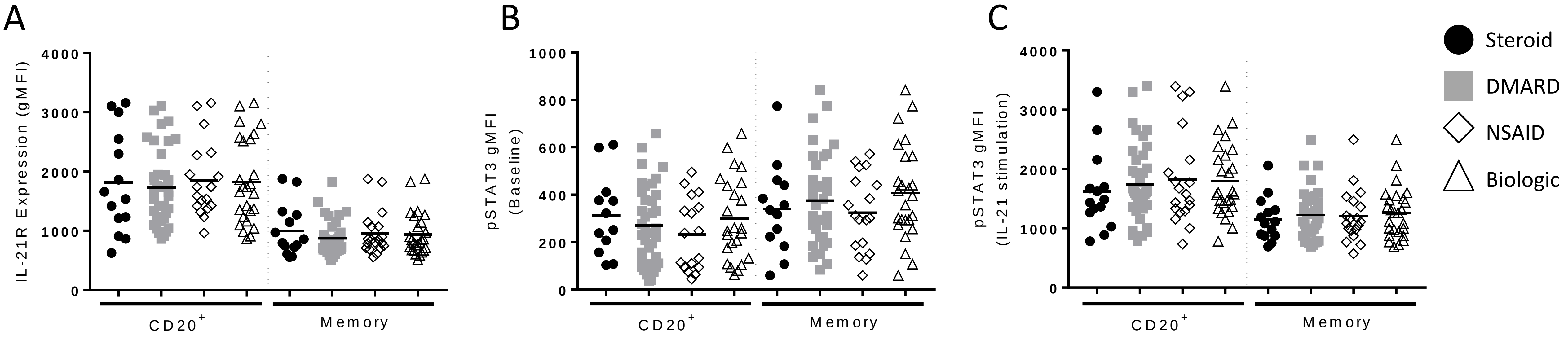

Supplemental Figure 5: No changes in IL-21R or pSTAT3 in RA patients categorized based on therapy. PBMC from RA patients were thawed and B cells (total and memory ($CD38^{lo}CD24^{hi}$)) were analyzed for IL-21R surface expression **(A)** pSTAT3 levels at baseline with no stimulation **(B)** pSTAT3 levels following a 45 minute IL-21 stimulation **(C)** Patients were categorized according to therapy: steroid, DMARD, NSAID and/or biologics while excluding those on rituximab.

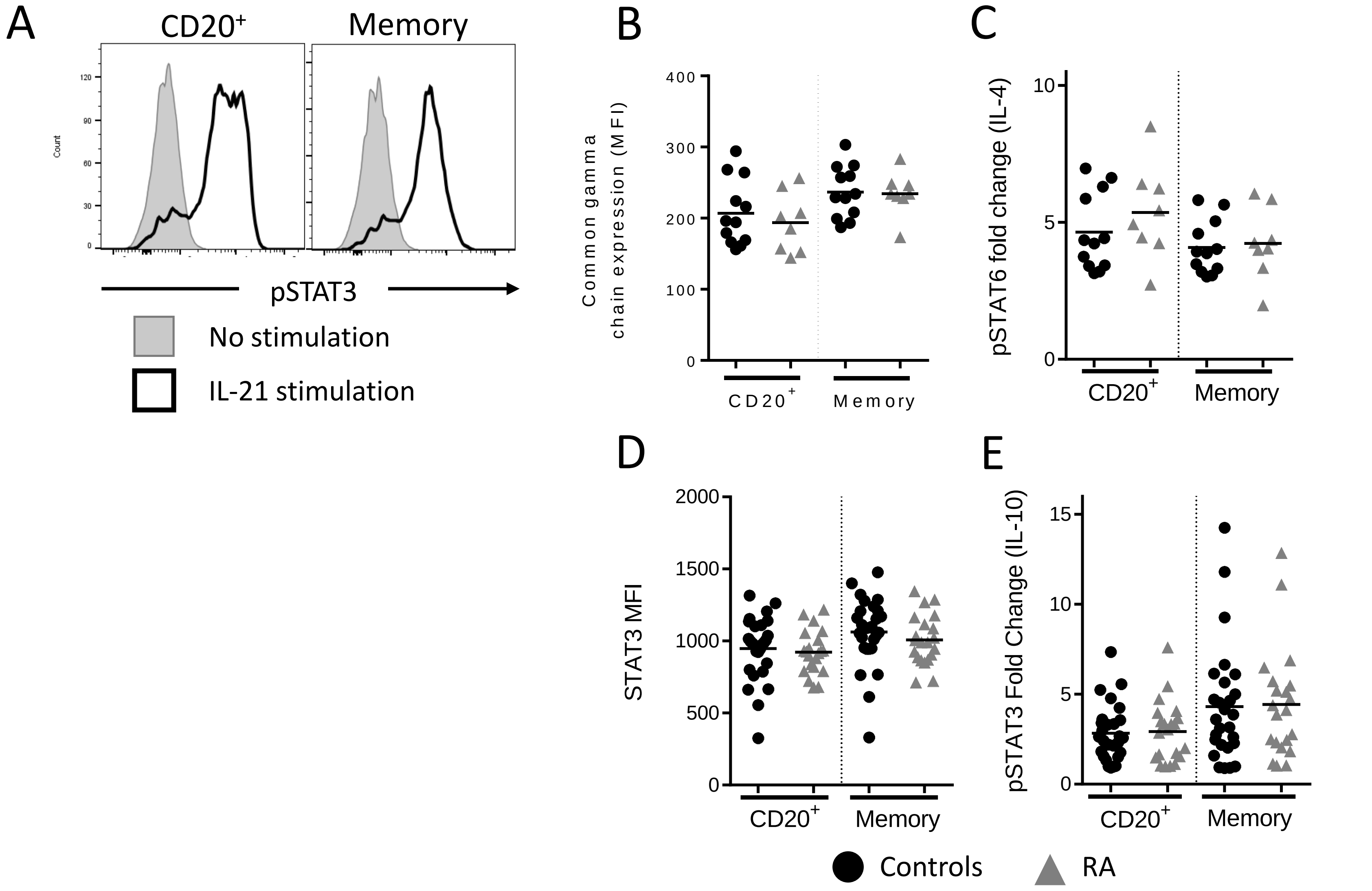

Supplemental Figure 6: No change in common gamma chain and STAT3 expression or IL-4 and IL-10 signaling in B cells from RA patients compared to controls. (A) Representative histograms of pSTAT3 levels with IL-21 stimulation (black line) or no stimulation (grey histogram) in CD20$^+$ and total memory B cells. **(B)** Baseline γ_C expression levels (C, n = 12; RA, n = 8), **(C)** pSTAT6 fold change levels after IL-4 stimulation (C, n = 12; RA, n = 8), **(D)** Baseline total STAT3 levels (C, n = 27; RA, n = 21) and **(E)** pSTAT3 fold change levels after IL-10 stimulation (C, n = 27; RA, n = 21) were determined in CD20+ and total memory B cells from control and RA patients. Significance was assessed by Mann Whitney U test in (B) – (E).

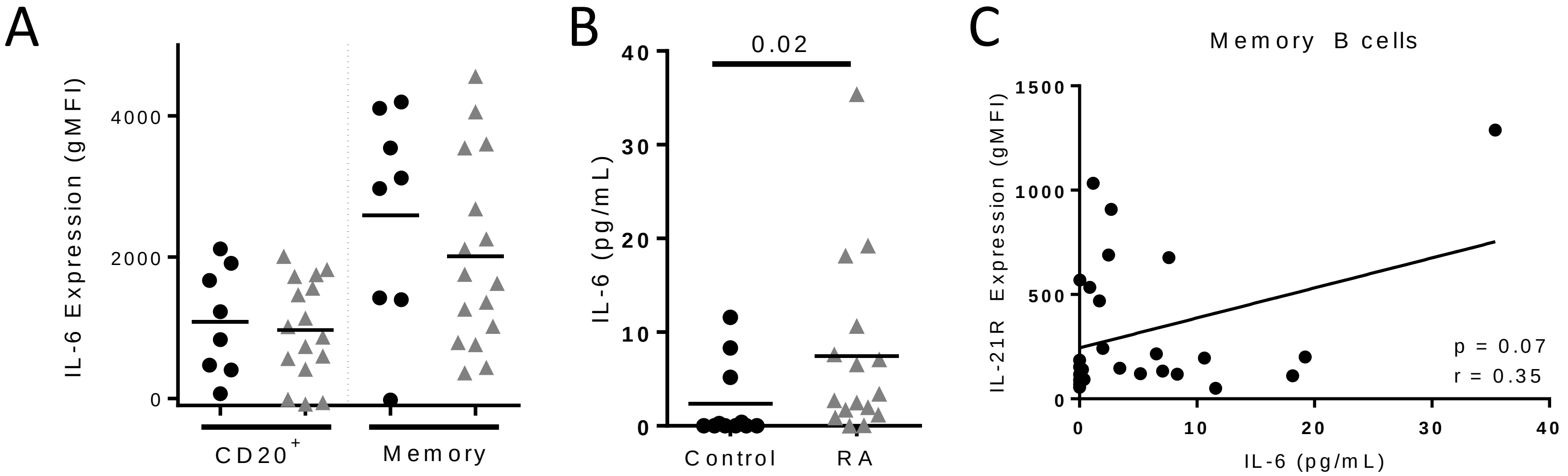

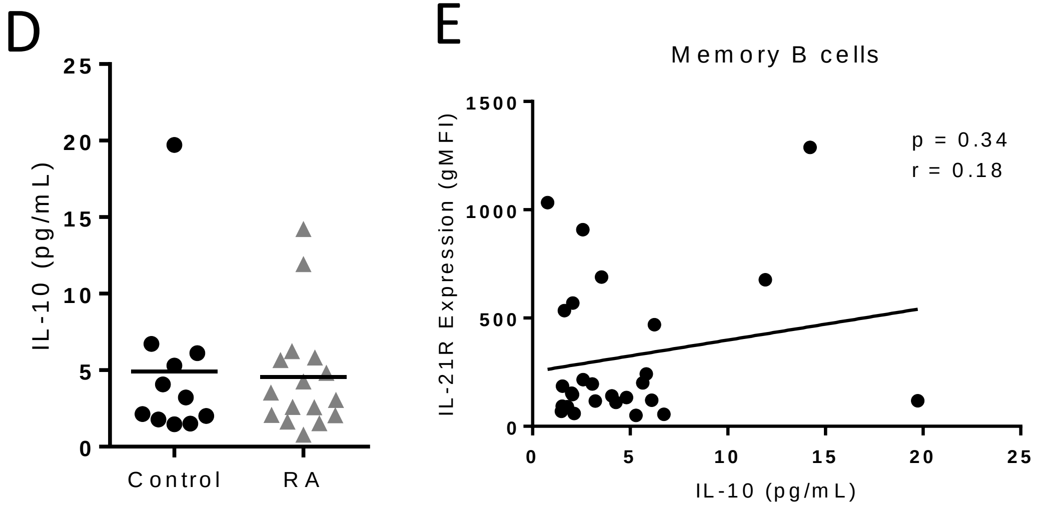

Supplemental Figure 7: No change in IL-6 expression when B cells are stimulated with PMA and Ionomycin. (A) PBMC were stimulated with PMA and Ionomycin for 4 hours after which intracellular staining was done to measure IL-6 production by $CD20^+$ and total memory B cells in control (n = 8) and RA patients (n = 16). **(B)** IL-6 levels were measured in the serum in controls and RA subjects which were **(C)** correlated to IL-21R levels on memory B cells. **(D)** IL-10 levels were measured in the serum in controls and RA subjects which were **(E)** correlated to IL-21R levels on memory B cells.

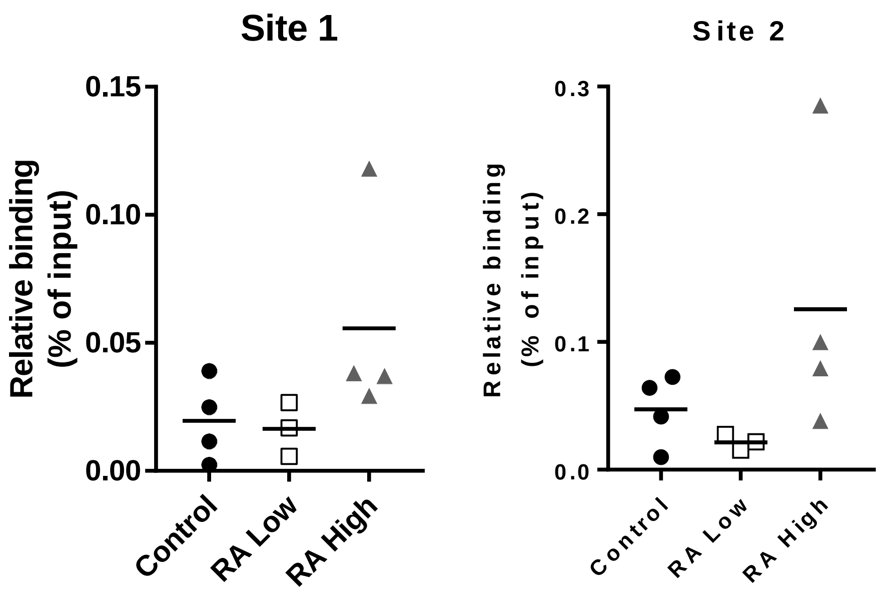

Supplemental Figure 8. No difference in SP1 binding in regions outside the *IL21R* gene. Total B cells were isolated from whole blood from RA patients and controls. SP1 binding to regions outside the *IL21R* gene were assessed by ChIP-qPCR analysis. The results are presented relative to input DNA (n = 4 for Ctrl, RA High) (n = 3 for RA Low).

A

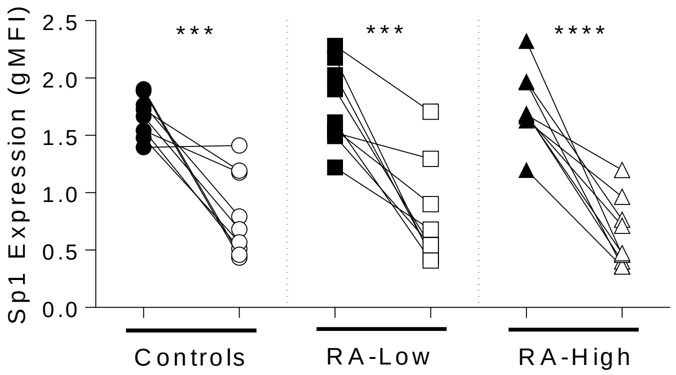

B

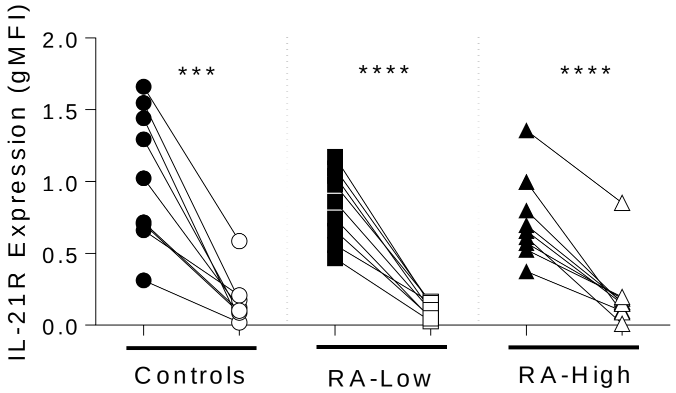

Supplemental Figure 9: Okadaic acid decreased SP1 levels and decreases IL-21R levels after a 24 hour incubation. Total PBMC from control and RA subjects were incubated with okadaic acid (OA) or a DMSO control for 24 hours. At 0 and 24 hours total (A) SP1 and (B) IL-21R expression levels were quantified in total B cells and normalized to the DMSO control. Analyzed by Student's paired t-test.
